# Developing inhaled protein therapeutics for lung diseases

**DOI:** 10.1186/s43556-020-00014-z

**Published:** 2020-10-30

**Authors:** Abigail A. Matthews, Pui Lai Rachel Ee, Ruowen Ge

**Affiliations:** 1 Department of Biological Sciences, Faculty of Science, National University of Singapore, 16 Science drive 4, Singapore, 117558 Singapore; 2 Department of Pharmacy, National University of Singapore, 18 Science Drive 4, Singapore, 117543 Singapore; 3 NUS Graduate School for Integrative Sciences and Engineering, 21 Lower Kent Ridge Road, Singapore, 119077 Singapore

**Keywords:** Inhalation, Proteins, Biologics, Drug delivery, Lung diseases

## Abstract

Biologic therapeutics such as protein/polypeptide drugs are conventionally administered systemically via intravenous injection for the treatment of diseases including lung diseases, although this approach leads to low target site accumulation and the potential risk for systemic side effects. In comparison, topical delivery of protein drugs to the lung via inhalation is deemed to be a more effective approach for lung diseases, as proteins would directly reach the target in the lung while exhibiting poor diffusion into the systemic circulation, leading to higher lung drug retention and efficacy while minimising toxicity to other organs. This review examines the important considerations and challenges in designing an inhaled protein therapeutics for local lung delivery: the choice of inhalation device, structural changes affecting drug deposition in diseased lungs, clearance mechanisms affecting an inhaled protein drug’s lung accumulation, protein stability, and immunogenicity. Possible approaches to overcoming these issues will also be discussed.

## Introduction

Biological drugs are revolutionising the treatment and management of many serious illnesses including cancer, autoimmune disorders, and rare genetic diseases, with about a third of all new drug approvals by the Food and Drug Administration (FDA) consisting of biological drugs [1]. However, over the past decades, the development of inhaled therapeutics for the treatment of respiratory diseases has largely been focused on small molecules (corticosteroids, β2 agonists, and muscarinic antagonists), with only one inhaled protein biologic drug Pulmozyme® being approved by the FDA to date [2]. In the treatment of lung diseases via inhalation therapy, biological drugs such as proteins/polypeptides offer many advantages over small molecule drugs. Proteins delivered via the pulmonary route could accumulate in the lungs while having a poor ability to traverse the air-blood barrier due to their large molecular weight. This would result in higher target site accumulation (airway epithelial cells, alveolar macrophages, neutrophils etc) and minimise systemic toxicity, as compared to small molecule drugs that would pass easily into the systemic circulation after reaching the lungs [2–4]. In addition, protein therapeutics display higher potencies (picomolar to femtomolar range) than small molecules (nanomolar range), as well as highly specific receptor binding to reduce off-target effects [5]. Notably, although peptide drugs (< 5 kDa or < 40–50 amino acids in length) share some of the characteristics with protein/polypeptide drugs such as both are composed of amino acids linked via peptide bond and both having high target specificity, most of peptide drugs are much smaller in sizes, conferring them with some distinct differences including less enzyme stability, higher tissue penetration ability etc. In addition, many peptide drugs harbour chemical modifications in the form of peptidomimetics and/or cyclization, and some have direct cell membrane penetrating ability. These peptide drugs are not covered in this review.

Protein therapeutics are conventionally administered via the systemic route, although this has proven to be an inefficient approach for drug delivery to the lung, not mentioning the additional danger of exposing the rest of the body vulnerable to toxicity [4, 6]. For instance, monoclonal antibodies (mAbs) are found at higher levels (500–10,000 times more) in serum than in bronchoalveolar lavage (BAL) fluid following intravenous administration, a trend that has been demonstrated in all species [4]. By the same token, protein therapeutics delivered via the airways also pass poorly from the lungs into the systemic circulation. For example, only low amounts of anti-VEGF-A G6–31 mAb (5.1%) and cetuximab (11%), an anti-EGFR mAb, were present in the serum after aerosol delivery in mouse models of lung cancer [7, 8]. This means that high concentrations of the protein drug can be attained in the lung via pulmonary delivery, suggesting that lower doses of inhaled protein can have an equivalent or even superior therapeutic effect for lung diseases when compared to the higher doses that would be needed from systemic administration [9]. Indeed, it was reported that the nebulised effective dose of AvidinOX-anchored biotinylated cetuximab was 1/25,000 of the intravenous effective dose in a mouse model of advanced metastatic lung cancer [10]. Although higher pulmonary levels of protein therapeutics can be achieved through inhalation compared to systemic administration, this could be offset by the short residence time of proteins in the lung compared to plasma. Proteins and antibodies are mostly cleared from the lungs within 24 h, while plasma half-lives of full-length antibodies following intravenous injection can reach 3 weeks and more [11]. Nevertheless, there are strategies that can be employed to increase the local residence time of protein therapeutics in the lungs, and these will be discussed in detail later on in this review. Besides improving pharmacokinetic and toxicity profiles of protein therapeutics, the inhalation route is non-invasive and allows for self-administration, which could improve patient compliance [4, 5, 12].

In 1993, Pulmozyme® (dornase alfa/deoxyribonuclease I), was introduced for the treatment of cystic fibrosis [9]. It has been almost three decades since then, and no other inhaled protein therapeutics for topical treatment of a lung disease has reached the market, despite the aforementioned advantages that inhaled proteins possess. Presently, and to the best of our knowledge, ten inhaled protein therapeutics are being assessed in clinical trials for the treatment of a range of lung diseases including asthma, cystic fibrosis, lung cancer, COPD and COVID-19 (Table 1) [13–18]. There are also protein therapeutics such as mAbs that are in the preclinical stages [19, 20]. In order to drive more of these therapies into clinical development and eventually to the market, it is crucial to take into account the challenges unique to the development of these agents into potential treatments so that they may be utilised successfully for pulmonary delivery. In this review, we will discuss the challenges in developing an inhaled protein therapeutic for lung diseases, as well as approaches that could help to circumvent these issues. The focus of this review is on the pulmonary delivery of protein drugs for local action in the lung, however, examples of inhaled proteins for systemic action will be mentioned wherever relevant.

**Table 1:** Inhaled protein drugs currently in clinical development for lung diseases

Protein	Drug Name	Sponsor(s)	Disease(s)	Development Phase	Type of inhaler	Clinical trial no./
						Reference
**Alpha-1 antitrypsin** (52 kDa)	Alpha-1 antitrypsin	Rabin Medical Center	Bronchiolitis obliterable syndrome (BOS) after lung transplantation	Phase II unknown status	–	NCT01394835
	Kamada-AAT	Kamada	Alpha-1 antitrypsin deficiency	Phase III ongoing	Mesh nebuliser (PARI eFlow® system)	NCT04204252
**Anticalin® protein (IL-4Rα antagonist)** (~ 18 kDa)	PRS-060/AZD1402	Pieris Australia Pty Ltd	Asthma	Phase I ongoing	–	NCT03574805
	–	AstraZeneca	Asthma	Phase I completed (2019)	DPI (Plastiape Monodose inhaler,) vs. Mesh nebuliser (Philips InnoSpire Go)	NCT03921268
**Anti –human thymic stromal lymphopoietin (TSLP) mAb fragment (Fab)** (46 kDa)	CSJ117	Novartis	Asthma	Phase IIb ongoing	–	NCT04410523
**Deoxyribo-nuclease I (DNase I)** (37 kDa)	AIR DNase™/ Alidornase alfa/ PRX-110	Protalix	Cystic fibrosis	Phase II completed (2017)	Vibrating mesh nebuliser (Philips I-neb® AAD system)	NCT02722122 [13, 14]
	Pulmozyme®	University Hospital, Strasbourg, France	Acute respiratory distress syndrome (ARDS)	Phase III ongoing	Vibrating mesh nebuliser (Aerogen® Solo device)	NCT03368092
	Pulmozyme®	Fondation Ophtalmolo-gique Adolphe de Rothschild	ARDS in coronavirus disease 2019 (COVID-19)	Phase III ongoing	Nebuliser	NCT04355364
	rhDNase	National Jewish Health	Neutrophilic asthma	Phase I/II ongoing	Nebuliser	NCT03994380
**Granulocyte macrophage colony-stimulating factor (GM-CSF)** (14 kDa)	rhGM-CSF	Dai Huaping China-Japan Friendship Hospital	Autoimmune pulmonary alveolar proteinosis (aPAP)	Phase II ongoing	–	NCT03316651
	Molgramostim	University of Giessin	ARDS	Phase II ongoing	–	NCT02595060
	Molgramostim	Savara Inc	aPAP	Phase III ongoing	Mesh nebuliser (PARI eFlow® system)	NCT03482752 [15]
	Molgramostim	Savara Inc.	Nontuberculous mycobacterial (NTM) infections	Phase II ongoing	Mesh nebuliser (PARI eFlow® system)	NCT03597347
	Sargramostim/Leukine®	Children’s Hospital Medical Center (Cincinnati)	aPAP	Phase I ongoing	–	NCT03006146
	Sargramostim/Leukine®	IRCCS Policlinico S. Matteo	aPAP	Phase II/III unknown status	Vibrating mesh nebuliser (AKITA^2^®)	NCT00901511 [16]
**Interferon beta-1a (IFNβ-1a)** (22 kDa)	SNG001	Synairgen Research Ltd.	Chronic Obstructive Pulmonary Disease (COPD)	Phase II ongoing	–	NCT03570359
	SNG001	Synairgen Research Ltd.	COVID-19	Phase II ongoing	–	[17]
**Interleukin-2 (IL-2)** (15 kDa)	Aldesleukin	MD Anderson Cancer Center	Lung metastases	Phase I/II ongoing	–	NCT01590069
**Lactoferrin (in combination with hypothiocyanite)** (80 kDa)	ALX-009	Alaxia SAS	Cystic fibrosis/Bronchiectasis	Phase I ongoing	Nebuliser	NCT02598999
**Sialidase** (46 kDa)	DAS181	Renmin Hospital of Wuhan University/Ansun Biopharma	Severe COVID-19	N.A. (Compassionate use) ongoing	Nebuliser	NCT04324489
	DAS181	Ansun Biopharma	Lower tract parainfluenza (PIV) infection/ COVID-19 substudy	Phase III ongoing	Vibrating mesh nebuliser (Aerogen® Solo device)	NCT03808922 [18]
	DAS181	Ansun Biopharma	COVID-19	Phase II/III ongoing	Vibrating mesh nebuliser (Aerogen® Solo device)	NCT04354389 [18]
	DAS181	Ansun Biopharma	Influenza/SAD-RV infections (including COVID-19)	Phase II ongoing	Vibrating mesh nebuliser (Aerogen® Solo device)	NCT04298060 [18]
**Tissue plasminogen activator** (70 kDa)	Alteplase	University of Michigan	Plastic bronchitis	Phase II ongoing	Nebuliser	NCT02315898

## Challenges and considerations in the development of protein therapeutics for local lung delivery

### Choice and limitations of drug delivery device

There are mainly three classes of inhalation devices namely dry powder inhalers (DPIs), nebulisers, and metered dose inhalers (MDIs). DPIs deliver drug as a solid aerosol, and powder formulations possess inherent stability and shelf life benefits [4, 21]. However, the temperature and shear stress during the manufacturing processes needed to produce powders (e.g. freeze drying, spray drying) could lead to protein degradation [21]. DPIs have been used for the marketed inhalable insulin formulations Exubera® (approved in 2006, but was discontinued after 1 year due to large device size, high pricing, and safety concerns) and Afrezza® (still commercially available) [22]. Moreover, DPIs have shown promising results in studies assessing their use for inhaled protein formulations. For example, Weers et al. (2019) showed that dry powder formulations of CSJ117 (anti-thymic stromal lymphopoietin (TSLP) mAb fragment) could achieve a total lung dose (TLD) of about 95% of the delivered dose (DD) with the use of particle engineering techniques such as via the introduction of surface corrugation through the addition of trileucine [23].

Nebulisers (jet, ultrasonic, and mesh) generate aerosol droplets from a liquid solution of the drug [4]. The first and only inhaled protein formulation approved for pulmonary delivery to date, Pulmozyme®, is administered via jet nebuliser. Nebulised formulations are less expensive to produce and test, because the manufacturing process for these formulations does not include extra drying steps. Nevertheless, prolonged storage of proteins in liquid solutions can lead to protein instability through degradation pathways (i.e. deamidation and hydrolysis), temperature and pH changes, and aggregation (through agitation of the aqueous carrier) [21]. Furthermore, across all nebuliser types, the process of nebulisation exposes the protein to physical stresses such as shearing forces and heat, as well as the large air-liquid interface (ALI) that could alter protein conformation and/or structure through denaturation, chemical modifications (oxidation, deamidation), and aggregation [4, 15]. Device-specific limitations such as the shear forces generated by jet nebulisers, and the temperature increases that occur in ultrasonic nebulisers, can also lead to protein degradation. Jet and ultrasonic nebulisers actually recycle 99% of the primary aerosol, and a molecule would typically be subjected to 10–15 cycles of nebulisation before leaving the nebuliser as a secondary aerosol [24]. This subjects the molecules to high shear stress in these devices, resulting in the denaturation of proteins, with the extent of protein denaturation and degradation varies depending on the characteristics of the individual protein [25]. For example, for jet nebulizer delivery of LDH and urease, there is a log-linear degradation with a fraction of protein degraded with every recirculation [26, 27]. In contrast, IgG and G-CSF has a rapid initial decline in native proteins in the first 5–10 min [26, 28]. For ultrasonic nebulizers, heating resulting from ultrasonic radiation in addition to aerosol recirculation generated various protein denaturation and degradation in different proteins [25]. For example, the degradation of LDH with ultrasonic nebulizer presented a sigmoidal progression instead, indicating different denaturation process and factors involved from jet nebulizers [29]. By comparison, vibrating mesh nebulisers employ single-pass technology, ensuring that there is no recirculation of droplets into the reservoir; they do not alter solution temperature, and produce less shear forces inside the drug reservoir during nebulisation, making them more suitable for the delivery of protein therapeutics [24–31]. In fact, several studies have reported that jet and ultrasonic nebulisers produce lower levels of activity, lower amounts of protein monomers (because of partial degradation), and more aggregates (with or without excipients). On the other hand, mesh nebulisers appear to maintain protein integrity to a greater extent than other nebulisers [32]. Safe aerosolisation with mesh nebulisers has already been demonstrated in several studies of labile drugs including proteins and mAbs [33–38]. The detailed designs and comparisons of various types of nebulizers have been reviewed previously and we will not elaborate further here [25, 30–32, 39].

Recently, nanoengineered particles using metal-phenolic networks (MPNs) with highly defined physical properties have been used to encapsulate both small molecule and macromolecules including proteins for pulmonary delivery via nebulisation. Intratracheal nebulization delivery of FITC-labelled bovine serum albumin (BSA, 65 kDa) in mice demonstrated that these capsules are biocompatible and biodegradable, showing > 85% of the capsules in the lung after 20 h, while only < 4% remaining after 30 days without causing obvious lung inflammation or toxicity. Although still in early stage of development, these MPN particles may revolutionize the nebulization delivery of protein drugs and provide a more protected environment for effective pulmonary delivery [40].

Moreover, new generation nebulisers are being developed such as surface acoustic wave (SAW) nebulisers and the more recent HYDRA (HYbriD Resonant Acoustics) nebulisers that provide new platforms for inhaled drug delivery. SAW use surface waves to generate aerosols which can preserve macromolecule integrity that has been shown to be efficient in aerosolising proteins [41]. HYDRA uses a hybrid combination of surface and bulk sound waves to generate the aerosol droplets, and can overcome the low nebulisation rate of SAW nebulisers and conventional nebulisers, while also avoiding the potential damage to proteins due to high shear (jet nebulisation) or cavitation (ultrasonic nebulisation). The first human lung deposition study using a prototype HYDRA nebuliser has been reported recently, indicating successful lung deposition of a radiolabelled small molecule [42]. It is probable that HYDRA nebulisers may be developed for pulmonary protein drug delivery.

MDIs deliver drug through an aerosol burst, and allow for the controlled delivery of specific amounts of drug to the lungs [21, 22]. However, as with nebulisers, the use of aqueous solutions is not ideal for protein storage [22]. There are also concerns that the hydrofluoroalkane (HFA) propellants used in MDIs could denature proteins [21, 43]. Despite this concern, there are examples of studies showing that proteins can remain stable in HFA-containing MDI formulations. Quinn et al. (1999) utilised Raman spectroscopy to analyse the secondary conformations of lysozyme in the HFA propellants tetrafluoroethane (HFA 134a) and heptafluoropropane (HFA 227), demonstrating that structural integrity of lysozyme was preserved in both HFAs, and that there is potential for proteins to be developed as MDI formulations without compromising their conformational stability [44]. Moreover, Liao et al. (2005) demonstrated that spray-dried lysozyme and catalase that were stabilised with excipients (sugars and/or 80% polyvinyl alcohol) and then stored in HFA 134a at room temperature for 6 months showed retention of biological activity [45].

When choosing an inhalation device, it is important to be cognisant of the fact that not all devices in the same category are equivalent. For instance, although it is generally accepted that there is minimal heating of the drug reservoir in vibrating mesh nebulisers, and that heating occurs to a lesser degree than in ultrasonic nebulisers, considerable temperature increases have been reported in some brands of vibrating mesh nebulisers (PARI eFlow®, AKITA^2^ APIXNEB®, and Aeroneb Go), with temperatures of up to 40°C being reached towards the end of nebulisation [24]. Therefore, it is essential to choose the inhalation device carefully, bearing in mind that the best device type is the one that confers the most stability to the protein drug formulation. Soft mist inhaler (SMI), which also generate aerosols from liquid, is the newest type of inhaler which does not use any propellent. As only one medicine Respimat uses SMI, its suitability for protein drug delivery is not clear.

Another factor to consider is the aerodynamic diameter of aerosol particles, which is critical to control where the particles will be deposited in the respiratory track after inhalation. To be therapeutically effective, the drug containing particles need to be deposited into the correct location within the respiratory track. For example, for therapeutics for COPD, drugs need to be delivered to the deep lung (the alveolar space) for which it requires the aerodynamic diameter of the particles to be between 1 and 5 μm. Larger size particles will generally be deposited in the oropharyngeal region and be ingested, while small particles < 1 μm may be exhaled during the next breathing cycle. Thus, suitable aerosol particle sizes need to be selected for precise drug delivery into the lung to enhance drug efficacy while simultaneously reducing harmful side effects [5].

### Structural changes in diseased lungs

The respiratory tract comprises of a series of branching airways, which can be categorised into two parts: the conducting zone and the respiratory zone. The conducting zone consists of the trachea, bronchi, bronchioles, and terminal bronchioles. The airway wall in the conducting zone is too thick for diffusion and this region does not contain alveoli. As such, no gas exchange takes place here, and the purposes of the conducting zone include transmitting air to the respiratory zone, as well as to warm, moisture and cleaning the inspired air. The respiratory zone consists of respiratory bronchioles, alveolar ducts, and alveolar sacs, and facilitates gas exchange between the air and the bloodstream. Alveoli can occasionally be found in the walls of the respiratory bronchioles, and are abundant in the alveolar ducts and alveolar sacs [45, 46]. Given the branched structure of the lungs, it is not only important to achieve high deposition rates, but also to obtain an appropriate deposition pattern for the respiratory disease in question i.e. the protein therapeutic would not only need to reach the lung, but would also need to reach the correct target site within the lung. For instance, a therapeutic for asthma would have to reach the large airway, as asthma mainly affects the bronchi, while a drug for emphysema in COPD would need to go deeper and reach the small airways of the lung because emphysema affects the alveolar region [47].

The amount and pattern of lung deposition is not only affected by the device and the characteristics of the inhaled drug (particle size and physicochemical properties of the formulation), but also by factors that are influenced by the specific disease state, including breathing patterns, lung geometry (i.e. airway diameter, number of alveoli) and structure, and nasal, oral, and pharyngeal anatomy [45]. These factors need to be considered, and if possible, alterations to the drug formulation can be made to address these issues. For example, in certain lung pathologies (for instance cystic fibrosis, COPD, and chronic sinusitis), the airway mucus becomes thicker. It has been reported that the thickness of the mucus layer ranges from 2 to 30 μm in normal lungs to more than 260 μm in cystic fibrosis and other obstructive airway diseases [48]. This presents a physical barrier that the protein drug would need to penetrate to reach its target site in the lung and exert its effects. The addition of anti-adhesive molecules (e.g. polyethylene glycol, PEG) in the formulation may help to promote the translocation of the protein drug through the thickened mucus, although it should be noted that the adhesive properties of PEG depend on PEG molecular weight (MW) [12]. While high MW PEGs display mucoadhesive properties, low MW PEGs are able to prevent mucoadhesion, with PEGs of MW up to 40 kDa able to provide effective mucus penetration [49–51].

Notably, as macromolecules, proteins have a relatively poor ability to penetrate the epithelial layer to reach the deep parenchyma lung. However, depending on the molecular weight and aerosol characteristics, a portion of the proteins would be able to reach the abluminal side of the epithelium or the air-blood interface in the thin alveolar wall, triggering local or even systemic immune signalling that may provide beneficial therapeutic effect in some cases [15].

### Clearance mechanisms in the lung

A good lung deposition pattern would be worthless if the protein therapeutic cannot withstand the lung’s clearance mechanisms. Inhaled proteins would be subjected to clearance by three mechanisms. The first clearance mechanism is mucociliary clearance (MCC), which is the coordinated beating of cilia lining the nasal cavity, trachea, and bronchi, in order to move the mucus towards the larynx/pharynx, thereby pushing dust, microorganisms, and insoluble particles that are trapped in the mucus out of the lungs and into the upper airways to eventually be swallowed [46]. The surface lining of the airways in normal lungs consists of an aqueous layer adjacent to the epithelium and a surfactant containing film layer at the air-liquid interface. The peri-ciliary aqueous layer has a relatively low viscosity, while the surfactant film layer is more viscous. The surfactant film plays an important role in the displacement of airway particles towards the epithelium where they will be immersed and retained. The extent of particle immersion depends on the surface tension of the film. The lower the surface tension, the greater the immersion of particles into the aqueous layer adjacent to the epithelium [52, 53]. It is possible that some protein monomers could quickly reach the stagnant aqueous layer and not be subjected to MCC. On the other hand, some protein monomers would become aggregated during the inhalation delivery process and the aggregates may stay with the surfactant film layer at the air-liquid interface for some time for MCC to take effect. Anti-adhesive formulations (achieved by using lower MW PEGs for example) could be used to circumvent MCC clearance of inhaled therapeutics, thus increasing their lung accumulation. The mucus-penetrating ability of such PEGs has already been demonstrated in multiple studies [12, 54–61].

The second clearance mechanism is macrophage uptake, which is the primary clearance mechanism in the alveoli. Proteins are taken up by alveolar macrophages in the deep lung via pinocytosis, and the uptake of particles is size dependent [5, 12, 62]. Large proteins (≥ 40 kDa) would have more time to be engulfed by alveolar macrophages by virtue of their slower transport and absorption across the alveolo-capillary barrier, while small proteins and peptides (≤ 25 kDa) are absorbed rapidly from the airspaces and thus, may not be impacted by alveolar macrophage uptake as much. In essence, pinocytosis by alveolar macrophages could become significant for macromolecules with MW > 40 kDa [62]. The use of excipients can help to reduce clearance of large proteins by alveolar macrophages. For example, Koussoroplis et al. (2014) showed that PEGylation conferred increased residence time to antibody fragments anti-IL-17A F (ab′)2 and anti-IL-13 Fab′ (unconjugated F (ab′)2 was 98 kDa and unconjugated Fab′ was 47 kDa), and that the effect was due to mucoadhesion as well as evasion of alveolar macrophage uptake [11]. Protein PEGylation may potentially also alter its deposition pattern in the respiratory track, due to changes in molecular weight, hydrophilicity etc. However, systemic analyses of the effect of PEGylation on protein deposition pattern in the respiratory track are still needed in order to know if a consistent deposition pattern and behaviour can be reached based on how PEGylation is achieved.

The third clearance mechanism is absorption into the systemic circulation. After deposition in the alveolar region, aerosol drug particles may dissolve in pulmonary epithelial lining fluid if the drug is water-soluble, and become available for systemic absorption and clearance [46]. For the purpose of topical lung treatment, the goal would be to minimise systemic absorption, which is greatly influenced by protein MW. The bioavailability of a protein after absorption from the lung decreases as protein MW increases. Small peptides are absorbed rapidly from the lungs with 20–50% of the bioavailability for subcutaneous injection [63]. Proteins with MW of 6–50 kDa exhibit moderate absorption, with bioavailability ranging from 10 to 40%, although it should be noted that pulmonary absorption studies in animals may lead to an overestimation of bioavailability. For example, systemic bioavailability after aerosol administration in animals for growth hormone (GH) and interferon α (IFNα) was 45% and 70% respectively, compared to only 3–10% in humans [63, 64]. Large MW antibodies (~ 150 kDa) are not significantly absorbed across the lung, and bioavailability is negligible (<< 10%) unless an active transport system is included [63]. Apart from high protein MW, the presence of obstructive lung diseases (e.g. asthma, COPD, cystic fibrosis) can also reduce systemic absorption and bioavailability of proteins and other drugs [46]. For example, Henry et al. (2003) reported that healthy subjects had significantly higher area under the curve (AUC) and mean maximum concentration (C_max_) after insulin inhalation than asthma patients, indicating that less insulin was absorbed into the systemic circulation in asthma patients [65]. In addition, Diderichsen et al. (2013) reported that the C_max_ of an inhaled long acting β_2_ agonist (PF-00610355) was found to be reduced by 31% and 52% for COPD and asthma patients respectively, compared to healthy volunteers [66]. Additional possibility for the lower systemic absorption of drugs in lung disease patients could be due to altered drug deposition pattern in the diseased lung, for which further studies are needed. Regardless the underlying mechanisms, effective local lung retention of protein drugs may not be a major issue for the successful inhalation treatment of obstructive lung diseases, since the protein can be relatively well retained in the lungs.

### Protein stability and immunogenicity

Inhaled protein therapeutics may undergo various degradation mechanisms during production, processing and/or storage. These degradation pathways may be physical (denaturation and non-covalent aggregation) or chemical (mainly covalent aggregation, deamidation, oxidation and/or glycation).

Denaturation is the result of physical stresses including low/high temperatures, high salt concentrations, organic solvents, and air/water or ice/water interfaces. Removal of the stressor may be spontaneously reversible (for some single domain proteins), but is usually irreversible for most of the larger multi-domain proteins [67]. Surface-induced aggregation is one of the common mechanisms of non-covalent aggregation, and one example of when it occurs is during the process of nebulisation [15, 67]. As most proteins are amphiphilic and surface active, they have a tendency towards adsorption at the ALI. Upon adsorption, conformation changes may occur, exposing hydrophobic residues to the interface to avoid contact with water, thus leading to aggregation and unfolding, which are the main factors contributing to protein instability [67, 68]. Chemical degradation of proteins (deamidation, oxidation, glycation) may also cause aggregation (either covalent or non-covalent) [67].

Aggregation has been extensively studied but chemical modifications have not, despite having implications on biological activity and immunogenicity [4, 15]. Aggregation can result from both physical and chemical pathways; therefore, it is useful to also evaluate chemical changes in inhaled proteins. Most studies assessing stability of inhaled protein formulations focus on formation of aggregates, while studies that also examine chemical changes are few and far between. One study did, however, consider chemical changes when evaluating the technical feasibility of delivering dornase alfa using perforated vibrating membrane devices for nebulisation. In this study, besides detecting protein aggregates, stability was also evaluated by measuring the percent deamidation of dornase alfa at Asn_74_ (the main chemical change for the protein), which was shown to be inversely proportional to dornase alfa potency [35]. Another study looked at methionine 59 oxidation [Met(o)] of nebulised insulin-like growth factor-1 (IGF-I), and how that correlated with aggregate formation and bioactivity. Highly aggregated samples displayed a complete loss of bioactivity, while samples with complete oxidation but minimal aggregation showed partial retention of bioactivity. Limited Met(o) formation and no aggregation was observed following delivery with air-jet or vibrating mesh nebulisers [36]. Bandi et al. (2019) conducted a study to compare the effects of deamidation and oxidation on interferon alpha-2a (IFNA2a), as deamidation of asparagine and glutamine residues, and oxidation of methionine residues are two of the most common chemical alterations that occur in pharmaceutical proteins that could compromise their efficacy and safety [68]. These findings revealed that deamidation destabilised IFNA2a and enhanced its tendency to aggregate under stressful conditions, and reduced its function to a greater extent than oxidation. This is the first study that quantitatively compared the effects between deamidation and oxidation of a therapeutic protein [68]. It would be a good strategy to conduct such studies early on in the development of therapeutic protein candidates in order to identify the chemical modifications that a particular protein would be susceptible to, and to test out various excipients that could resolve specific stability issues.

The protein therapeutic also needs to remain stable after reaching the lung, which can be challenging due to the high numbers of serine proteases and aminopeptidases present in the lung mucosa [69–72]. These proteases could degrade protein drugs even before they reach their target sites within the lung. The use of appropriate excipients in the formulation such as PEG, could help to enhance protein resistance to proteolysis by these lung proteases. For example, Zhang et al. (2014) evaluated the stability of fibronectin (FN) preferentially PEGylated at lysine residues using different MW PEGs [2 kDa (PEG2), 5 kDa (PEG5) or 10 kDa (PEG10)] against the protease α-chymotrypsin. They showed that PEGylation protected FN from proteolysis and that PEG MW positively correlated with proteolytic stability (i.e. after 30 min of proteolysis, 4%, 34%, 43% and 65% of the starting amounts of native FN, FN-PEG2, FN-PEG5 and FN-PEG10 respectively were remaining) [73].

One must also be aware of the possibility of protein aggregates forming in the lungs. Lasagna-Reeves et al. [74] demonstrated that mice exposed to inhaled insulin (Exubera®) in a chamber twice daily for 1 week developed amyloid aggregates of insulin in both the proximal and distal airways, as well as the lung parenchyma (epithelium and muscle layer of the bronchi, bronchioles, and in the alveolar lining cells). The formation of insulin aggregates coincided with a significant decrease in respiratory flow rates, and also with caspase-9 activation. Previous studies investigating the link between changes in pulmonary function and inhaled insulin use focused on formation of anti-insulin antibodies, or pulmonary inflammation and subsequent airway remodelling, but none of the published works before this looked at insulin aggregation in the lungs as a contributor to pulmonary dysfunction after inhaled insulin use [74]. Indeed, Exubera® was reported to cause cough, dyspnea, increased sputum and epistaxis [75]. This example highlights the possibility of inhaled proteins forming aggregates in the lungs, and thus the need for toxicity testing and safety studies examining this possibility to be done early on in the development of an inhaled protein candidate, during preclinical studies.

In addition, proteins and other macromolecules have the potential to induce immunogenicity, with the production of anti-drug antibodies (ADAs) as the main immune response [5]. The development of ADAs in patients can alter pharmacokinetics, drastically reduce efficacy, and can also lead to severe adverse events or even lethal consequences [76]. Immunogenicity is also linked to protein stability, as the presence of aggregates can render the protein immunogenic. As aggregates are typically composed of denatured molecules, they would exhibit no or decreased activity, but at the same time, aggregates are usually immunogenic leading to ADAs with important clinical implications [4]. Aggregates are believed to be recognised and processed via non-specific uptake by antigen presenting cells and specific uptake by B cells. They may unmask neo−/cryptic/repetitive epitopes, and these differences may influence the mechanism by which they activate the immune system [76].

## Approaches to address the challenges in formulating inhaled protein therapeutics

### Usage of excipients

Currently, only a few excipients have been approved by the FDA for inhalation due to a dearth of toxicological studies for inhaled excipients [15, 67]. There is also very limited number of excipients that are approved by FDA for biologics, rendering formulators limited choices to improve protein formulations when excipients are searched on the FDA’s Inactive Ingredient Database Guidance. Furthermore, very few novel excipients have been investigated for biologic products; most are cyclodextrin-based excipients [77]. As such, there is a need for more extensive toxicity testing to identify novel excipients for pulmonary delivery. For excipients already known to increase protein stability, a trial and error approach needs to be taken in determining their suitability for a particular protein formulation, as an excipient may work for one protein but not for another for various reasons including sequence differences [15]. Excipients that are commonly used in liquid formulations (nebulisers and MDIs) include buffering or pH adjusting agents, and surfactants, and those that are commonly used in dry powder formulations (DPIs) include sugars, polyols, and amino acids [67, 78].

Buffering or pH adjusting agents such as sodium chloride, sodium citrate, hydrochloric acid, sodium hydroxide, and citric acid, are added to maintain the pH of the formulation. It is important to choose the right buffering agent at an appropriate concentration, as most proteins in solution only remain stable within a narrow pH range. Different buffer systems and concentrations can also affect the aggregation pattern of proteins [67]. Kim et al. [79] analysed the stability of a fusion protein, etanercept (marketed Enbrel®), with changing pH and buffer concentrations. Increasing the pH of etanercept from pH 6.6 to 8.6 resulted in a decrease in protein size and increase in aggregation. Under high buffer concentrations (30 mM Tris buffer), changes in protein size was reduced and irreversible aggregation was not observed, while in lower buffer concentrations (10 mM Tris buffer), larger aggregates (~ 1 μm) were observed across the pH range [79].

Surfactants (polysorbates, sorbitan esters, oleic acid, and soy lecithin) are frequently used to prevent aggregate formation, and they work by displacing protein molecules from the ALI [46, 62]. Polysorbates are the most commonly used surfactants, and are already being used to preclude aggregation in formulations of intravenously administered antibodies [34]. Polysorbate 80 has been reported to lead to stabilisation in various inhaled protein formulations including those for granulocyte-colony stimulating factor (G-CSF), lactate dehydrogenase (LDH), tissue plasminogen activator (t-Pa) and aviscumine (recombinant mistletoe lectin) [68]. The ability of polysorbates and other surfactants to stabilise a protein and hinder aggregate formation is contingent on the protein-to-surfactant ratio. Respaud et al. [34] examined the effects of various antibody and surfactant (polysorbate 20) concentrations to optimise the protein-to-surfactant ratio for a nebulised antibody formulation. The authors determined that high concentrations of either surfactant or protein could minimise the formation of medium and large-sized aggregates, without significantly affecting the volume mean diameter (VMD) of the aerosol cloud, ensuring suitability for inhalation. Therefore, including surfactants and raising protein concentration to enhance the stability of inhaled protein formulations is a viable strategy, although it should be noted that this approach needs to be evaluated and optimised for each drug and device pairing being developed into an inhaled protein formulation [34].

Sugars (sucrose, trehalose, raffinose and lactose) and polyols (mannitol) stabilise proteins through the preferential hydration of proteins via steric repulsion of sugar/polyol molecules from the native protein [4, 68]. Lactose is often used as a drug carrier in DPIs, however, it may not be suitable for proteins because it is a reducing sugar, and it could interact with amino groups in proteins (Maillard reaction) [67]. On the other hand, non-reducing sugars such as sucrose, trehalose and raffinose would not undergo the Maillard reaction with proteins, and thus could be used as alternatives to lactose [81]. Sellers et al. [82] demonstrated that sucrose could help to improve the stability of a dry powder formulation of LDH. Supercritical fluid (SCF) drying of LDH without excipients lead to irreversible loss of activity (only 15% recovered after rehydration). Inclusion of 10% (w/w) sucrose during dehydration lead to an increase in activity recovered (to ~ 60%), and there was almost complete retention of activity when polysorbate 20 was added in addition to sucrose [82]. Trehalose and raffinose are currently not approved for any administration routes, but have been evaluated in experimental studies with promising results. For instance, Ógáin et al. [81] incorporated lysozyme into nanoporous microparticles of trehalose and raffinose. Lysozyme showed good retention of specific activity after storage for 12 weeks at either 4°C (98.2 ± 7.1% for lysozyme:trehalose and 99.1 ± 7.1% for lyzosyme:raffinose) or 25°C (92.5 ± 7.1% for lysozyme:trehalose and 90.8 ± 7.1% for lyzosyme:raffinose) [81]. Mannitol was used as an excipient in the formulation of Exubera® (Table 2) [80].

**Table 2:** Excipients used in marketed inhaled protein formulations

Product	Device type	Active Pharmaceutical Ingredient (API)	Excipients
Pulmozyme® (approved 1993)	Jet nebuliser	Dornase alfa (DNase I)	sodium chloride, calcium chloride dihydrate
Exubera® (approved 2006, discontinued 2007)	DPI	Insulin human	mannitol, sodium citrate, glycine, sodium hydroxide
Afrezza® (approved 2014)	DPI	Insulin human	fumaryl diketopiperazine, polysorbate 80

Small amino acids (histidine, arginine, alanine, glycine, lysine, isoleucine) are also used as stabilisers, and they work by the “water substitution mechanism” in which the amino acids hydrogen bond with the protein during drying to preserve the native protein structure in the dried state [4, 83]. Ajmera and Scherlieβ [84] screened different amino acids and their combinations for their ability to stabilize catalase during spray drying. When various ratios of arginine, glycine and histidine were mixed with catalase, some formulations were able to maintain close to 100% catalase activity [84]. Despite encouraging results in studies such as this one, there is a lack of data on the local toxicity of the various amino acids following inhalation, which could limit their use. However, as they are endogenous substances, they may not present major safety issues for local lung delivery [67, 85].

The polyol, PEG, could be used for both liquid and powder formulations of inhaled proteins. Small MW PEGs (< 10 kDa) are often used as excipients in oral, intravenous and nasal formulations. Larger PEGs (up to 40 kDa) may be used in PEGylated biopharmaceuticals, and safety testing for these formulations are done during development on a case-by-case basis [86]. PEGylation is a commonly used method to enhance solubility and stability, as well as to decrease immunogenicity of bioactive drugs including but not limited to proteins, peptides, antibody fragments, and enzymes, and is achieved by the covalent or noncovalent conjugation of PEG to the biomolecule [87, 88]. PEGylation can also help to reduce clearance and increase lung accumulation and residence time of inhaled protein therapeutics. For instance, conjugation of a PEG chain to two antibody fragments (anti- IL-17A F (ab′)2 and anti-IL-13 Fab′) increased their levels in mouse lungs following intranasal administration. Forty-eight hours post-administration, levels of unconjugated antibody fragments in the lungs had dropped to 10% and 14% of the original deposited dose of F (ab′)2 and Fab′ respectively, while this value was 40% for both PEGylated fragments [11]. Furthermore, conjugation of a PEG chain to an anti-IL-17A Fab’ antibody fragment increased pulmonary retention in all three species tested (mice, rats, and rabbits) following intratracheal administration. Unconjugated fragments were cleared from the lungs within 24 h while large amounts of PEGylated fragments still remained for up to 48 h [89].

### Encapsulation in carriers

The two biggest challenges in developing particle systems for pulmonary drug delivery are to maintain colloidal stability during aerosolisation and to achieve high delivery efficacy. Encapsulation of proteins in carriers could provide multiple benefits such as protection from enzymatic degradation and specific targeting to the site of action through targeting ligands [5]. Furthermore, carriers may also be used to provide sustained drug release, accumulating in the lungs and releasing therapeutic levels of the protein drug over extended periods of time. This would enhance efficacy while averting peaks in local drug concentrations that could cause pulmonary toxicity [90]. Proteins, including insulin, calcitonin, and IgG, have already been loaded into various carriers such as microparticles, liposomes, and solid lipid nanoparticles [90, 91]. Indeed, Afrezza® uses Technosphere® technology, in which fumaryl diketopiperazine (FDKP), an excipient added into the formulation, self-assembles into microspheres, entrapping the insulin. Upon reaching the alveolar zone of the lung, the Technosphere® particles rapidly dissolve in the pH-neutral environment and release the insulin for systemic absorption [75]. Although this approach has not been extensively explored for topical lung delivery of proteins, and more work needs to be done on the use of carriers for the purpose of systemic delivery of proteins through the lungs, some promising results have been reported that support further development of this approach. Tawfeek et al. [92] encapsulated a model mucinolytic enzyme, α-chymotrypsin (which is very sensitive to unfolding and formulation conditions), in a novel biodegradable PEG-co-polyester microparticle carrier. The encapsulated α-chymotrypsin exhibited retention of enzymatic activity and the results indicated suitability of the carrier for potential use in the delivery of macromolecules as DPI formulations for the treatment of lung diseases [92]. In another study by Osman et al. [93], various surface modifications were made to DNase I loaded microparticles using different excipients in order to provide higher lung deposition, enzyme stability and biological activity. Surface modifying the microparticles with polyglutamic acid (PGA) or dextran was found to provide high inhalation indices (emitted fraction (EF), respirable particle fraction (RP), and effective inhalation index (EI)) and increased mucolytic activity in cystic fibrosis sputum. This could be explained by the resulting surfaces of the particles after modification with PGA (rough dented surfaces) or dextran (dimpled surfaces). Compared to spherical particles with similar physical properties, corrugated particles have surface asperities that could reduce the true contact area between particles, decreasing powder cohesiveness and enhancing aerosol performance [93].

Advancements in drug-loaded capsules for pulmonary delivery have been made in both inhalable dry powder or liquid drug formulations [94–96]. For dry powder drug particles, precise control of the particle size has been reported using the Particle Replication In Nonwetting Templates (PRINT) technology [97, 98]. For control of aerodynamic particle size in liquid aerosols such as in nebulized liquid formulation, the recently reported MPNs have presented promising possibilities. MPN-based drug-loaded capsules with highly defined physical properties can be generated for both macromolecular protein drugs and small molecule chemical drugs [54]. These new developments may transform inhalation drug delivery in the near future.

One drawback with the use of carriers is their rapid uptake by alveolar macrophages [99, 100]. Phagocytosis of carriers by alveolar macrophages can result in fast clearance and reduced residence time, limiting the therapeutic efficacy of the carrier-associated drug. This would be an issue for the treatment of chronic lung diseases such as asthma and COPD, where the goal of using a carrier system would be to achieve controlled and continuous drug release over an extended period of time. However, various formulation design strategies may be employed to reduce the uptake of particulate carriers by alveolar macrophages including modulation of particle size, shape, surface charge and surface coating [101]. Studies on the use of various polymer coatings demonstrate reduced alveolar macrophage uptake of coated carriers. For example, Jones et al. [102] showed that respirable microspheres coated with dipalmitoyl phosphatidylcholine (DPPC; a major component of lung surfactant) were able to significantly reduce phagocytic uptake by NR8383 in cultured alveolar macrophages compared to uncoated microspheres. The uptake of DPPC coated microspheres was found to be only 24.1 ± 7.86%, 31.9 ± 3.74% or 36.6 ± 3.66%, of the uptake of uncoated microspheres for ratios of 5, 10 or excess microspheres per NR8383 cell respectively [102]. Furthermore, Shen et al. [103] demonstrated that surface coating of hydrogel nano- and microparticles with PEG showed significantly reduced uptake by alveolar macrophages both in vitro (in MH-S cells) and in vivo (in mice) compared to unPEGylated particles of the respective size. At 24 h post-dose, the fold difference between PEGylated and unPEGylated 80 × 320 nm, 1.5 μm, and 6 μm particles in bronchioalveolar lavage fluid (BALF), was 1.5, 3.4 and 3.7 respectively [103]. On the other hand, drug-loaded particles may be advantageous for anti-tuberculosis drugs as efficient uptake of drugs into alveolar macrophages could potentially enhance the drug’s efficacy to kill the parasitic *Mycobacterium tuberculosis* that hide inside the cells [104].

If the usage of a carrier is to be included in the protein formulation, it should be noted that the formulation (i.e. combination of protein and carrier) would need to be optimised together with the choice of device, as the chosen carrier may not work well with all inhalation device types. For instance, liposomes may be delivered to the lungs either by dry powder inhalation or nebulisation of a liposome suspension. However, nebulised solutions of liposomes may cause instability as nebulisation has been reported to disrupt liposomal structure, leading to the release of loaded drug. These issues can be avoided with the use of dry powders of liposomes instead [90]. Hence, although this review has presented a general overview for the various aspects of protein formulation design (such as choice of device, excipients), it is important to test out the formulation and device together to determine which combination works best.

## Concluding remarks

**Fig. 1:**
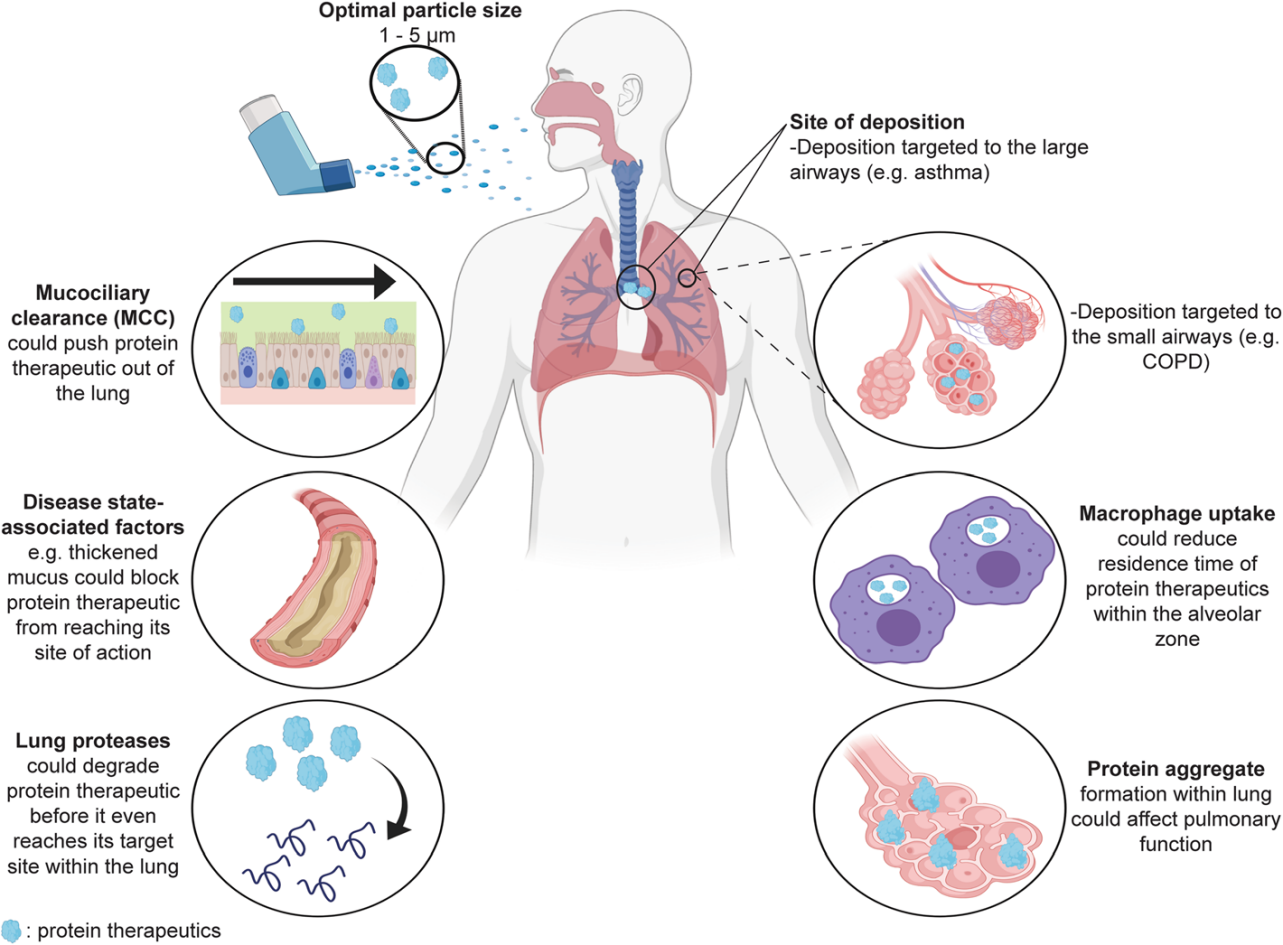
Major challenges for targeting inhaled protein therapeutics to the lungs for local action. Targeted deposition sites within the lung (large or small airways) will vary depending on the specific lung disease being treated. (Created with BioRender.com)

This review analyses the various obstacles that an inhaled protein drug would need to overcome in order to reach the lungs and exert its therapeutic effects. These obstacles include the physical and chemical stresses experienced by the protein during production/storage/aerosolisation, the need to overcome mucociliary clearance and physical barriers arising from disease conditions in order to reach target sites within the lung, and the need to remain stable in spite of the presence of abundant proteases, and to evade clearance by alveolar macrophages after reaching the lungs (Fig. 1). All of these threats to the integrity of the protein need to be carefully considered, so that pre-emptive measures can be taken while designing the protein formulation to ensure its therapeutic efficacy. Nevertheless, although the information provided here may serve as general considerations in developing pulmonary protein therapeutics, empirical testing of the formulation together with the device should still be performed to determine the best combination for a particular protein.

Several key areas will require further investigation in order to support the development of more successful inhaled protein therapies, and maintaining the stability of the inhaled protein is of paramount importance. Firstly, more studies could look at other instability issues beyond protein aggregation. In depth studies on the specific chemical modifications that a protein would be susceptible to, such as the one conducted by Bandi et al. (2019), could be carried out on therapeutic protein candidates so that they may be developed into stable and effective treatments [68]. Moreover, the scarcity of FDA-approved excipients for inhaled therapeutics further limits drug developers, and expanding this list through increased toxicological testing of new excipients would provide more options for formulation design. Finally, innovative approaches such as the use of novel carrier systems should be employed for the purpose of topical lung delivery, as carrier systems could greatly enhance the stability and pharmacokinetic profile of proteins. These approaches would greatly benefit the field of pulmonary drug delivery, and will ultimately allow more inhaled protein therapeutics to reach the clinic.

## Data Availability

NA.

## Abbreviations

API: Active pharmaceutical ingredient
ADAs: Anti-drug antibodies
AUC: Area under the curve
BSA: Bovine serum albumin
COPD: Chronic obstructive pulmonary disease
DD: Delivered dose
DPPC: Dipalmitoyl phosphatidylcholine
DPIs: Dry powder inhalers
EI: Effective inhalation index
EF: Emitted Fraction
FN: Fibronectin
FDA: Food and Drug Administration
FDKP: Fumaryl diketopiperazine
G-CSF: Granulocyte-colony stimulating factor
HYDRA: HYbriD Resonant Acoustics
HFA: Hydrofluoroalkane
IGF-I: Insulin-like growth factor-1
LDH: Lactate dehydrogenase
MDIs: Metered dose inhalers
MPNs: Metal-phenolic networks
Cmax: Mean maximum concentration
MW: Molecular weight
mAbs: Monoclonal antibodies
MCC: Mucociliary clearance
PRINT: Particle Replication In Nonwetting Templates
PEG: Polyethylene glycol
PGA: Polyglutamic acid
RP: Respirable particle fraction
SMI: Soft mist inhaler
SCF: Supercritical fluid
TSLP: Thymic stromal lymphopoietin
TLD: Total lung dose
VMD: Volume mean diameter
